# Assessing health professionals’ perception of health literacy in Rhode Island community health centers: a qualitative study

**DOI:** 10.1186/s12889-020-09382-1

**Published:** 2020-08-26

**Authors:** Mary L. Greaney, Sherrie F. Wallington, Sankeerth Rampa, Vivian S. Vigliotti, Carol A. Cummings

**Affiliations:** 1 Department of Health Studies, University of Rhode Island, Kingston, RI 02881 USA; 2 The George Washington School of Nursing & Milken Institute School of Public Health, Washington, DC 20006 USA; 3 School of Business, Rhode Island College, Providence, RI 02908-1991 USA; 4 Robbins Institute for Health Policy & Leadership, Baylor University, Waco, TX 76798 USA; 5 Department of Health & Physical Education, Feinstein School of Education and Human Development, Rhode Island College, Providence, RI 02908-1991 USA

**Keywords:** Community health centers, Health literacy, Focus groups, Qualitative research, Social determinants of health

## Abstract

**Background:**

Limited health literacy is linked with poor health behaviors, limited health care access, and poor health outcomes. Improving individual and population health outcomes requires understanding and addressing barriers to promoting health literacy.

**Methods:**

Using the socio-ecological model as a guiding framework, this qualitative study (Phase 1 of a larger ongoing project) explored the interpersonal and organizational levels that may impact the health literacy levels of patients seeking care at federally qualified community health centers (FQCHCs) in Rhode Island. Focus groups were conducted with FQCHC employees (*n* = 37) to explore their perceptions of the health literacy skills of their patients, health literacy barriers patients encounter, and possible strategies to increase health literacy. The focus groups were audio-recorded and transcribed, and transcripts were coded using a process of open, axial, and selective coding. Codes were grouped into categories, and the constant comparative approach was used to identify themes.

**Results:**

Eight unique themes centered on health literacy, sources of health information, organizational culture’s impact, challenges from limited health literacy, and suggestions to ameliorate the impact of limited health literacy. All focus group participants were versed in health literacy and viewed health literacy as impacting patients’ health status. Participants perceived that some patients at their FQCHC have limited health literacy. Participants spoke of themselves and of their FQCHC addressing health literacy through organizational- and provider-level strategies. They also identified additional strategies (e.g., training staff and providers on health literacy, providing patients with information that includes graphics) that could be adopted or expanded upon to address and promote health literacy.

**Conclusions:**

Study finding*s* suggest that strategies may need to be implemented at the organizational-, provider-, and patient- level to advance health literacy. The intervention phase of this project will explore intervention strategies informed by study results, and could include offering health literacy training to providers and staff to increase their understanding of health literacy to include motivation to make and act on healthy decisions and strategies to address health literacy, including the use of visual aids.

## Background

Establishing good health requires having the skills to access and understand needed health services and information, complete health forms, effectively communicate with health care providers, and apply knowledge and skills to enhance health [1]. About 80 million people in the United States (US) have limited health literacy, which is concerning since limited health literacy is linked with poor health behaviors—including smoking, being physically inactive, and consuming a poor diet [2, 3]—and poor health outcomes [4–7]. Limited health literacy also is associated with limited access to health care [8, 9]. Older adults, racial and ethnic minorities, individuals with lower income, and individuals with less education are more likely to have limited health literacy skills [10]. Health literacy has been defined and conceptualized in many ways, such as having the ability or skills needed to access, comprehend, assess, and use health information and services for decisions and actions that support health on both an individual and community level [10, 11]. Obtaining and maintaining good health is dependent on one’s capacity to carry out these skills and the cognitive and social skills needed for action [12, 13]. In clinical settings, health literacy has been viewed as the capacity to comply with directives received from health care providers and as an asset that can be improved through health promotion efforts [14, 15].

Federally qualified community health centers (FQCHCs) in the United States are mandated to provide primary care in underserved communities regardless of an individual’s ability to pay. There are approximately 1400 FQCHCs funded by the US Department of Health and Human Services’ Health Resources & Services Administration (HRSA) [16]. The Affordable Care Act created the Community Health Center Fund and provided $11 billion over a five-year period to support the operation, expansion, and construction of health centers. This increased funding has contributed to an increase in the number of community health centers and in the number of patients being served, with one in 12 people across the United States receiving care at FQCHCs [16].

Given that FQCHCs provide care to underserved populations and the relationship between health literacy, health behaviors, and health status, understanding health literacy levels of patients seeking care at FQCHCs is important since this information can inform strategies at the patient-, provider-, and health-center levels to address and promote health literacy. Improving individual and population health outcomes requires addressing barriers to health literacy through a multitude of public health initiatives [17–19]. One important strategy to promote health literacy is to provide training to health care professionals [17]. Patients’ health literacy capacity or skills are affected by the patient-provider interaction [20], which alludes to the importance of providing training and education on health literacy to health professionals. The health care workforce should understand the scope of health literacy and be proficient in assessing patient health literacy [21] because providers’ communication skills affect both patient and population health [22]. Professional education efforts to address the impact of limited health literacy should address cultural differences that exist between health care providers and patients [23]. The health care workforce will continue to be faced with barriers to patient care if they do not seek training or are not trained to understand health literacy and cultural health beliefs and practices of a US population that continues to become more diverse [23, 24]. There is a growing realization that the characteristics of health care organizations impact individual and population health and “that health literacy makes it easy for people to navigate, understand, and use information and services to take care of their health” [25]. Thus, the purpose of this qualitative study was to explore the perceptions of health care providers and staff about health literacy, their patients’ health literacy, strategies used to address health literacy, and possible ways to address health literacy within their FQCHC.

## Methods

### Study design

This study is Phase 1 of a mixed-methods project entitled *Assessing Health Literacy in Rhode Island and Building Health Literacy Skills in Vulnerable Populations and in the Health Care Professionals Who Serve Them.* Phase 1 is a qualitative study, while Phase 2, which is ongoing, is a cross-sectional survey assessment of health literacy skills of patients receiving care at *FQCHCs* in Rhode Island. Phase 3 will include a pilot test of organizational-level interventions to address and promote health literacy.

### Theoretical framework

The socio-ecological model (SEM) provided the theoretical framework for this qualitative study [26]. The SEM posits that an individual’s health status, related behaviors, and/or choices are shaped by factors at the intrapersonal, interpersonal, organizational, community, and policy levels. In terms of health literacy, intrapersonal-level factors can include an individual’s capabilities, personal resources, and coping skills in the face of challenging health information and/or instructions, while interpersonal-level factors can include support of family and friends, and communication with health care providers and staff. Organizational-level factors related to health literacy include communication and support within organizations such as community health centers, which are dependent on the neighborhood or greater community’s available resources [26, 27]. Policy-level factors associated with health literacy include factors such as funding for and mandating of health interpreters and health advocates, policies regarding the use of plain language communications, and so forth. The SEM has been used to understand a wide variety of health factors, beliefs, and behaviors, from confidence in the human papillomavirus (HPV) vaccine [28], to access to human immunodeficiency virus (HIV) treatment [29], to students’ food choices while at school [30], to getting the flu shot [31], to being physically active [32, 33], and to sedentary behavior [34]. The current qualitative study focused on exploring interpersonal and organizational levels of SEM that may impact patients at FQCHCs in Rhode Island. Specifically, focus groups were conducted with health care providers and staff at the FQCHCs (an interpersonal level of interaction) who work with the patients and their families.

### Setting

This study was conducted in three sites of two FQCHCs in urban and suburban areas of Rhode Island. One of the FQHCs provides health care services to residents in and around a city in Rhode Island and the other serves residents in central, northern, and southern Rhode Island. *S*tudy staff met with the chief medical officer or operating officer (individuals who are responsible for medical supervision and overall regulation of the FQCHC at which they are employed) of each site and discussed the entire project (Phases 1–3), including time commitment and eligibility requirements. Eligibility requirements for FQCHC included (a) being a FQCHC located in Rhode Island, (b) allowing data collection to take place onsite, and (c) agreeing to participate in an intervention once developed. After deciding to participate, the medical officer or operating officer the participating FQCHCs then contacted the site directors at each site, who were the primary contacts for recruiting providers and staff to participate in the focus groups. Site directors manage the day-to-day operation of the site but do not need to be trained medical professionals.

### Focus group eligibility

Participant eligibility for the focus groups included being employed at one of the participating FQCHCs as a health care provider (e.g., nurse, medical assistants, doctors) or a staff member (e.g., billing personnel, community health workers) who interacts with patients and speaks English. Eligibility was limited to health care providers and staff because it was necessary for focus group participants to have interactions with patients.

### Recruitment

Purposive sampling was used to recruit focus group participants. Purposive sampling is widely recommended since focus group discussion relies on the ability and capacity of participants to provide relevant information [35]. As such, we specifically sought out FQCHC health care providers and staff to share relevant information about their individual, patient, and organizational health literacy experiences. The site directors facilitated recruitment by sharing the flyers with both health care providers and staff. The flyers asked interested providers and staff to contact their site director if they were interested in participating. The site director and study staff worked together to schedule the focus groups at times convenient to the health centers.

### Focus group administration

Focus groups were held at each participating FQCHC during the workday, with interested participants allowed to attend by their supervisors. Focus groups were led by moderators trained in qualitative research methods using a semi structured discussion guide with prompts that explored participants’ perception of health literacy, their patients’ health literacy, strategies used to address health literacy, and possible ways to ensure patients understand offered health information. The discussion guide (Supplementary file 1) was piloted at a site of one of the participating FQCHCs and revised before use in the current study. These data are not included in this study.

The moderator informed focus group participants that to protect anonymity the recordings and transcripts would not be shared, although selected de-identified quotes would be included in reports and papers. Refreshments were served, and all participants participated in the informed consent process and provided a signed informed consent before the start of each focus group. The focus groups lasted approximately 60–75 min and were audio-recorded.

To ensure confirmability, which concerns the aspect of neutrality and that the interpretation is grounded in the data and not based on the researcher [36], the moderators engaged in active listening and paraphrased statements back to the participants noting agreement and disagreement. Verbal active listening strategies commonly used in qualitative research include paraphrasing, reflecting, interpreting, summarizing, and checking perceptions [37]. These strategies provide the participants with a “mirror” so to speak in which to examine the message, expand on it, correct it, and reflect on the implication [38]. In addition, the data were independently coded by two researchers (VV and SW) using NVivo.

### Data analysis

The focus group recordings were professionally transcribed, and the de-identified transcripts were individually coded using NVivo version 12 [39] by two members of the study team (SFW, VSV). Data were entered into NVivo to prepare for coding. Based on the health literacy literature and the focus group guide, an initial codebook was developed, with new codes being added as needed. The initial coding process involved reading the transcripts one by one and marking keywords in context on each transcript. During open coding, the constant comparative approach [40, 41] was used to group the codes into categories and identify themes.

Axial coding was then applied to look at the interrelationship of themes. Repetitive words and phrases were placed together under selected categories, or *nodes*. Nodes allow the researcher to gather related material in one place to look for emerging patterns and ideas. Each node was based on the codes developed by the researchers, which were guided by the literature and theoretical framework. Intercoder reliability between the two investigators, assessed using Kappa statistics, was 1.0. The Kappa coefficient is a measure of agreement between raters or measurement procedures for categorical data, and a value of 1.0 indicates perfect agreement [42].

## Results

Two FQCHCs were approached to participate in the study, and both agreed to participate. Five focus groups with 37 FQCHC employees were conducted at three different sites in December 2018. About half of the participants (40.5%, *n* = 15) were Hispanic, 89.2% (*n* = 33) were female, and 51.4% (*n* = 19) had a four-year college degree or more (see Table 1). Eight themes were identified during analysis, and they are presented below with illustrative quotes.

**Table 1:** Demographic characteristics of focus group participants (*n* = 37)

	***n*** (%)
**Gender**	
Female	33 (89.2)
Male	4 (10.8)
**Age Group**	
18–24 years	6 (16.2)
25–34 years	13 (35.1)
35–44 years	12 (32.4)
45–54 years	2 (5.4)
55+	4 (10.8)
**Hispanic, Latino, or of Spanish Origin**	
Yes	15 (40.5)
No	22 (59.5)
**Race**	
Asian	3 (8.1)
Black or African American	6 (16.2)
White	22 (59.5)
Other	2 (5.4)
Missing	4 (10.8)
**Education**	
High school graduate or the equivalent	3 (8.1)
Some college	2 (5.4)
Trade/technical/vocational training	7 (18.9)
Associate degree	6 (16.2)
Bachelor degree	11 (29.7)
Master degree, professional degree, doctorate	8 (21.6)

### Theme 1: definition of health literacy

Most participants broadly defined health literacy when asked to define the term. These definitions focused on having the knowledge and ability to live a healthy lifestyle. As one participant explained, “I feel like health literacy is having knowledge about terms that have to do with health. That could be medical, mental health, and having an understanding about how exercise, diet, medications, how everything contributes to health.” In addition, many participants defined health literacy in terms of individuals having the ability to understand their health conditions. One participant stated, “It’s [health literacy] the patient’s understanding of their diagnosis, their level of understanding [what’s] being told to them and [understanding] it.”

### Theme 2: patients’ sources of health information

When asked where patients find health information, many participants predominantly described media-based sources (television, internet, and radio). One participant commented, “A lot of patients get their information from the internet or from TV.” Similarly, another focus group participant stated, “[Patients will say], ‘I saw this on TV,’ especially, ads, some of the lawsuit ads. Patients just say, ‘I stopped taking that medication because on TV, they said it will kill me.’”

A number of focus group participants also spoke of patients obtaining information from friends, family members, and others in the community. One participant explained that patients may get information at “the bodega, where they buy all their antibiotics.”

### Theme 3: perception of patients’ health literacy

Participants felt that many patients seeking care at their FQCHC had limited health literacy skills. Many participants felt that their patients’ limited health literacy made it difficult for them to access, understand, and evaluate health information, including the validity and reliability of health information. One participant stated, “Sometimes they [patients] don’t have the accessibility, or they don’t know how to navigate the internet, or sometimes you do teach them, but they just don’t retain things, so it’s hard for them.”

Most participants attributed their patients’ limited health literacy to limited education, which affected the ability of some to understand and process information about their health conditions. As one participant explained,

Some of our patients are not well educated. They never had biology. You start talking about the heart and the valves and all these things. They don’t know what you’re talking about. Not only do doctors talk in their own language, we all know that, but if you don’t know the basics about a cardiovascular system or how diabetes affects the body, if you have no clue then you got an awful lot of learning that you’ve got to do to bring you up to the point of “this is what’s going on.” … That’s a lot of information.

### Theme 4: challenges associated with limited health literacy

In discussing challenges, many participants covered both challenges caused by limited health literacy skills and the difficulty of patients overcoming limited health literacy. Participants spoke of patients having personal barriers, such as fears of illness or children being taken away, and these fears resulted in patients feeling overwhelmed and impacted their ability to overcome challenges associated with health literacy. As one participant said, “Even some [patients] are concerned that if we do find something in the screening, one of the patients said, ‘I just don’t want you to take away my kids.’”

Additionally, a number of participants spoke of practical barriers that made it challenging for some patients to act on health information. Noted practical barriers included patient’ emotions overriding learning, cost and access for lifestyle changes, care managers missing information, and patients’ literacy level and retention of information. Participants also discussed the organizational barrier of limited time (discussed in Theme 5 below), which limited patient-provider interactions in which providers could provide explanations and address possible misunderstandings.

### Theme 5: limited time is a barrier to addressing health literacy

Most participants spoke of having limited time to interact with patients due to patient volume being a barrier to addressing health literacy and ensuring that patients understand their diagnoses and next steps in their treatment, including use of medications. As one participant said, “I think that sometimes it’s the time that we need … sometimes we don’t have the time to explain everything to the full capacity.” Similarly, another participant explained,

They’re [patients] given 20-min apps [appointments], and it’s sometimes a lot to go over in that 20 min. We might be rushing just to get to the next patient, not really sitting down and making sure [the current patient] can understand the matter or the problem.

Some participants spoke of limited patient-provider interactions as increasing patients’ reliance on nurses, translators, and medical assistants for information. One participant vocalized possible risks associated with this practice and said,

It’s risky because we don’t necessarily have all of the information or [are] equipped with accurate information, and then we also have to think about our scope of practice and what’s outside of our scope of practice because we don’t want to offer advice or education without consulting the doctor first, but those things are hard to be coordinated.

### Theme 6: strategies employed to address limited health literacy

Focus group participants spoke of themselves and others at their FQCHC utilizing new learning approaches to address limited health literacy and increase patients’ understanding of their health conditions and treatment plans. Specifically, many participants spoke of using simple language, presenting information in a variety of ways to increase patient understanding, and using visuals and interactive media (e.g., apps). As one participant explained, “I like when they [doctors] have pictures, really clear pictures because a lot of our patients don’t read.” Participants also spoke of working to ensure patients understand what providers say during their appointments. One participant noted,

When we go into appointments with patients, we do the same thing. If the doctor’s not or the provider’s not giving them information in a way that they understand, we kind of rework it so that they do understand it or give them a breakdown of what’s going on with them medically and [what] has to be done to kind of clear it up.

In addition, a number of participants mentioned trying to spend additional time with patients who need assistance. Participants also spoke of preparing patients before their doctor’s appointments and speaking with patients after their appointments to review what was discussed and to discuss next steps. As one participant explained, “A lot of people here work with patients directly, so we explain a lot of what our providers are trying to explain to them, but I think in more simpler terms [to] help them understand.” Similarly, another participant said,

“Afterwards [after appointment], we have time to drive it home or meet with them in the waiting room after to review what the doctor said and to also advocate, to make sure [of] what their concerns are.”

Moreover, several participants highlighted speaking with providers before appointments to inform them if patients had difficulty comprehending health-related information. One participant said,

I think as a nurse care manager, we can really bridge that gap a little bit by talking with the doctors and having that communication with them. … I try to prepare the patient a little bit for that visit with the doctor.

Similarly, another participant spoke of informing providers when a patient has limited health literacy.

I think that it’s just having a conversation with the provider. To say, “Hey, this patient does have an issue and has shared their concerns with us,” but they do have a difficult time understanding information that we can give them.

Participants also noted that they work with patient advocates and encourage patients with limited health literacy to have family members attend their appointments.

### Theme 7: organizational culture impacts ability to address differences in patients’ and providers’ health literacy

Most participants felt that organizational changes could help bridge the gaps of health literacy between some patients and providers. For example, some participants mentioned that providers may need education and training on how to transform complex ideas into simpler ones. A number of participants also felt that the FQCHC should encourage all providers to use plain language and to make the use of plain language the norm. As one participant explained,

Training would be to have them [doctors] talk in layman’s terms. I feel like in my experience, most doctors, they go to school for years and years and years, and they’re extremely intelligent. They just don’t have that heart of being empathetic … they don’t think like that. They don’t think to speak to a patient, maybe they don’t even know how, so it would be an intense training. It would be like going to social work school and learning all those skills.”

Several participants did mention that many providers already were using plain language when meeting with patients. One participant responded,

I think the providers that I work with, especially the ones that have been here for a while, are very used to speaking the language of our patients. … It’s almost kind of baked into the conversation, like we’re all going to describe that symptom that way to each other and to the patient because it’s how the patient told us about it.

Most participants across the focus groups felt that patients at their FQCHC were treated equally and respectfully and highlighted that the health center’s employees come from varied backgrounds, which helped to promote the equal treatment of and respect for patients from varied backgrounds. As one person stated,

[Providers/staff] have tons of training—on transgender, on working with disillusioned patients or Spanish-speaking patients. We have a variety of employees who also come from different backgrounds, which also helps.”

### Theme 8: suggestions for ameliorating the impact of low health literacy

In contemplating strategies that could improve health behaviors and outcomes by addressing low health literacy skills, participants’ suggestions generally fell into three categories: (a) organizational-level strategies, (b) provider-level strategies, and (c) improving health education efforts. Organizational-level strategies that were discussed included promoting teamwork to ensure patients understand shared information and hiring additional nurses. One participant remarked,

If money fell out of the sky, we could have a lot more nurse managers—that would be huge. They’re nurses, they’re capable, they’re smart, they’re caring, they understand disease, they work closely with the doctors. … It’s a wonderful thing, but they’re expensive.”

Another organizational-level strategy that several participants discussed was creating centralized databases for health-related information that staff can share with patients. These participants felt these centralized databases would reduce time spent trying to find information and would ensure that patients were offered culturally appropriate materials at the right reading level. One participant commented,

I think it would be better if it was centralized where [the health center] would almost have a pool of all the pre-diabetes, diabetes, thyroid, where now each nurse just researches and goes on whatever website and posts their own information.

The provider-level strategies that participants discussed were focused on offering providers health literacy training, educating providers about patient needs and context, and increasing use of plain language. In addition, participants spoke of the need to offer patients problem-solving support, education, and follow-up. One participant stated, “Even if there were some sort of cultural competency that they could do to kind of help them [doctors] understand more of the plight of low-income people [it would help].”

One suggestion for health education materials included improving them at the patient level for better comprehension; such changes should focus on literacy levels and should include graphics to represent information. As two participants commented,

Have the materials in their [patients’] language. That would help for starters.

I think that using actual objects, visual objects [would help]. Getting back to diabetes, I have a plate, we talk about the plate and I have the plate. So for the patient to actually to see that and breaking it up and let’s divide it and then I have a handout that goes with it. We discuss it and let them take it home.

## Discussion

This qualitative study used the SEM as a guiding framework to explore the impact interpersonal and organizational levels may have on the literacy levels of patients seeking care at FQCHCs. Using focus groups and purposive sampling was an appropriate approach to explore perceptions of health care providers and staff regarding health literacy. All focus group participants were versed in health literacy and defined health literacy as having the ability to understand and access health information needed to lead a healthy lifestyle. Participants also defined health literacy as having the ability to understand any medical conditions and being able to access and understand information to promote health and healthful behaviors. Participants regarded patients’ health status as being impacted by their health literacy. This finding is important since health care providers and medical staff who realize the importance of health literacy may be more likely to adopt strategies to address limited health literacy. It also is important to assess contextual factors because individuals with low levels of health literacy may encounter barriers at the individual, interpersonal, community, and organizational level that impact their ability to access health care and supportive services [43, 44].

In the current study, focus group participants spoke of patients obtaining health-related information from media-based sources (television, internet, and radio) and from family members and friends. Internet use in the United States is high and ranges from 73% of adults aged 65+ to 97% among adults aged 18 to 29 [45]. Participants also spoke of patients having difficulty determining whether accessed information is reliable. A study conducted in 2017 found that 61% of respondents would like training on how to use online resources to find trustworthy sources, with differences identified by race/ethnicity (75% of Hispanics, 70% of blacks, 55% of whites) [46]. Taken together, these findings suggest that offering training on evaluating information sources may be a useful strategy to promote health literacy at the participating FQCHCs.

Results of the current study, as guided by the theoretical framework of the SEM, provide considerable insights into perceptions around health literacy at the interpersonal and organization levels. One of the organizational-level strategies discussed in the focus groups was the idea of having a centralized health information database, including websites, that could be used to provide patients with vetted materials in simple language. A qualitative study conducted in a federally qualified health clinic in Missouri revealed, as part of their needs assessment, that individuals who participated in the in-depth interviews (patients, non-clinical and clinical support staff, providers, administrators) thought that such a resource would be useful [47]. As part of the intervention, a library of plain language diabetes self-care materials for patients was created. Interviews conducted at the end of the intervention revealed that time was likely a barrier to creating additional health topic libraries and that providers often continued to select traditional materials (versus selecting from the library) for patients, although selecting materials with greater awareness of plain language [47]. If a database is created at the participating FQCHC, it will be important to convey the utility of the database to providers and also ensure that time is allotted to create and update the database.

Research suggests that 40–80% of medical information is forgotten immediately, and much of the retained information is inaccurate, although written information is better remembered than spoken information [48]. However, processing and acting upon written information can be challenging for people with limited health literacy skills. Participants in the current study felt that pictures and visual aids are important tools for conveying information. Barros and colleagues [49] conducted a literature review examining the use of pictographs in health care and included studies in the review if they (1) examined the use of pictographs for health education in patient; (2) were written in English, Portuguese, or Spanish and published between January 1960 to March 2009; and (3) were available in searched data bases [49]. In total, 24 studies were eligible and included in the review. Of these studies, 12 were conducted in Africa, four were conducted in Europe, four took place in North America, two were conducted in South America, one was conducted in Asia, and one study did not include study location. Of the studies meeting eligibility criteria, 23 employed pictographs focused on medication use [49]. About half (51.4%) of the studies were considered effective, and 29.1% did not report effectiveness, which was assessed by whether the examined articles included information stating that the pictograms resulted in the recall of previously provided information, increased understanding, or adherence of prescribed pharmacotherapy [49]. The use of pictographs or pictorial information is a useful strategy to convey information to individuals with limited literacy levels [50–53]. Possible intervention strategies to be explored in Phase 3 of the current study will focus on implementing organizational-level changes such as providing training on assessing health literacy and utilizing strategies such as providing patients needed information via pictures and using simple language. Building these skills allows the health care workforce to better communicate with patients and assist them with accessing essential information, services, processing questions, and making healthy decisions. Other organizational-level strategies such as making strategic plans for initiating and spreading health literate practices, establishing a health literacy workforce and supporting structures, raising health literacy awareness and training staff system-wide have been cited in the literature [25].

This study has several limitations which limit generalizability, including a small sample size (*n* = 37). Additionally, the focus groups were at work with the participants’ colleagues thereby possibly biasing comments shared. Most participants were women and it is possible that women and male employees of the FQCHC have had different interactions with patients that would results an alternative assessment of discussed topics. Further, the study was conducted at only two FQCHCs and the participating FQCHCs are likely supportive of improving and addressing health literacy since they allowed the focus groups to be conducted during the workday. Lastly, the participating FQCHCs were located in urban/suburban areas of RI, and prior research suggests that there are differences in health literacy by rural-urban status, with health literacy rates being lower in urban areas although this difference may be dues in age, gender, race/ethnicity, education, and income [49]. We believe we demonstrate trustworthiness in our study through documented and systematic description or of planning and methodological processes. Collectively, we believe that our IRB approval, theoretical framework, methodological procedures including moderators use of paraphrasing back to participants, and NVivo analysis provides an indication of the trustworthiness of this work [54].

## Conclusion

Participants in the study offered comprehensive definitions of health literacy and spoke of patients’ health literacy levels having implication on health status. Most participants spoke of their own strategies to address health literacy and organizational-level strategies to address health literacy. Study finding*s* suggest that strategies may need to be implemented at the organizational-, provider-, and patient- level, study to advance health literacy. Study findings also suggest that future interventions strategies at the participating FQCHCs could include offering health literacy training to providers and staff to increase their understanding of health literacy to include motivation to make and act on healthy decisions and strategies to address health literacy such as asking clarifying questions during appointments, helping patients remember medical information by using explicit categorization techniques and supporting spoken information with written or visual aid material such as infographics. All of these will support patient’s ability to make and act on health promoting decisions.

## Supplementary information

**Additional file 1: Supplementary file 1.** Discussion guide.

## Data Availability

At the start of the focus groups, the moderator informed focus group participants that to protect anonymity the recordings and transcripts would not be shared, although selected de-identified quotes would be included in reports and papers. Therefore, the datasets generated used for the current study are not publicly available as this option was not presented to the focus group participants.

## Abbreviations

FQCHC: Federally qualified community health center
RI: Rhode Island
SEM: Socioecological model
