# Supplementary material for: Assessing health professionals’ perception of health literacy in Rhode Island community health centers: a qualitative study
Source: BMC Public Health. 2020 Aug 26;20:1289. doi: 10.1186/s12889-020-09382-1 (PMC7448344; doi:10.1186/s12889-020-09382-1)
Supplement: Supplementary file 1 — Additional file 1: Supplementary file 1. Discussion guide. [file 12889_2020_9382_MOESM1_ESM.docx]

**Focus Group Questions**

1. Could you explain what you believe health literacy is?

Health literacy is the degree to which individuals have the capacity to obtain, process, and understand basic health information and services so that they may make appropriate health decisions.

Probe: What are your thoughts about health literacy?

1. What are some ideas for new learning approaches around health literacy for the healthcare workforce and patients?
2. Do you think patients with Medicaid demonstrate health literacy skills linked with: a) accessing information; b) understanding information and deriving meaning; c) interpreting and evaluating medical information; and d) making informed decisions?
3. How well do you think patients with Medicaid at this health center:
   1. Understand what the doctor/providers says to them?
   2. Assess the reliability of health information from mass media?
   3. Access mental health information for stress or depression?
   4. Understand information on medicine packaging?
   5. Participate in health promotion activities?
4. How can ______ health center build health care professionals capacities to strengthen patient health literacy skills?  Who is responsible for this?
5. Do you feel health provider’s written and spoken communication has sufficient clarity and quality at this health center?

Probe: What are some ideas to improve written and spoken communication?

1. Do you feel any printed materials at your health center helps patients learn about medical conditions, medical providers or insurance, or their health conditions?

Probe: How could printed materials be better organized for patients?

1. What is the biggest difficulty you feel patients face when trying to find needed health information?

Probe: Where do you think they look for health information?

1. When patients are filling out medical information is there anyone within the healthcare facility to help them?

Probe: Does anyone offer to help patients better understand certain medical information?

1. How do you believe patients with different backgrounds are treated?
2. Does anyone take someone else with them to medical appointments?

Probe: Do you think it is helpful when patients have someone with them when going through a medical experience or making medical decisions?

1. Now, I want to go around the room and give each of you an opportunity to briefly comment on anything discussed today that you may not have had a chance to respond to.
